# A balanced game: chicken macrophage response to ALV-J infection

**DOI:** 10.1186/s13567-019-0638-y

**Published:** 2019-03-06

**Authors:** Min Feng, Tingting Xie, Yuanfang Li, Nan Zhang, Qiuyuan Lu, Yaohong Zhou, Meiqing Shi, Jingchen Sun, Xiquan Zhang

**Affiliations:** 1 Guangdong Provincial Key Laboratory of Agro-animal Genomics and Molecular Breeding, College of Animal Science, South China Agricultural University, Guangzhou, China; 2 Key Lab of Chicken Genetics, Breeding and Reproduction, Ministry of Agriculture, Guangzhou, Guangdong China; 3 Division of Immunology, Virginia-Maryland Regional College of Veterinary Medicine, University of Maryland, College Park, MD USA

## Abstract

Avian leukosis virus subgroup J (ALV-J) infection can cause tumors and immunosuppression in infected chickens. Macrophages play a central role in host defense against invading pathogens. In this study, we discovered an interesting phenomenon: ALV-J replication is weakened from 3 hours post-infection (hpi) to 36 hpi, which was verified using Western blotting and RT-PCR. To further investigate the interaction between ALV-J and macrophages, transcriptome analysis was performed to analyze the host genes’ function in chicken primary monocyte-derived macrophages (MDM). Compared to the uninfected control, 624 up-regulated differentially expressed genes (DEG) and 341 down-regulated DEG at 3 hpi, and 174 up-regulated DEG and 87 down-regulated DEG at 36 hpi were identified in chicken MDM, respectively. ALV-J infection induced strong innate immune responses in chicken MDM at 3 hpi, instead of 36 hpi, according to the analysis results of Gene Ontology and KEGG pathway. Importantly, the host factors, such as up-regulated *MIP*-*3α*, *IL*-*1β*, *iNOS*, *K60*, *IRG1*, *CH25H*, *NFKBIZ*, lysozyme and *OASL* were involved in the host defense response during the course of ALV-J infection. On the contrary, up-regulated *EX*-*FABP*, *IL4I1*, *COX*-*2*, *NFKBIA*, *TNFAIP3* and the Jak STAT pathway inhibitors including *CISH*, *SOCS1* and *SOCS3* are beneficial to ALV-J survival in chicken macrophages. We speculated that ALV-J tropism for macrophages helps to establish a latent infection in chicken MDM from 6 to 36 hpi. The present study provides a comprehensive view of the interactions between macrophages and ALV-J. It suggests the mechanisms of defense of chicken macrophages against ALV-J invasion and how ALV-J escape the host innate immune responses.

**Electronic supplementary material:**

The online version of this article (10.1186/s13567-019-0638-y) contains supplementary material, which is available to authorized users.

## Introduction

Avian leukosis virus subgroup J (ALV-J) is an oncogenic retrovirus, primarily inducing neoplastic diseases and reproduction problems in infected chickens. It is well known that ALV-J causes enormous economic loss in the global poultry industries. To date, there are no vaccines or treatments to protect against ALV-J infection. Since little is known about the interaction between ALV-J and the host, current strategies are focused on ALV-J eradication. RNA viruses are prone to mutations. In contrast to the virus, the host does not change quickly. It is therefore an enticing strategy to try to overpower ALV-J by finding ways to make chickens less permissive to viral replication.

Studies concerning host innate and adaptive immune responses to ALV-J are in their infancy [1]. Macrophages are found in all tissues and have a well-defined role in host responses against viral infection [2]. However, macrophages are susceptible to infection for human immunodeficiency virus (HIV) [3], dengue virus [4], and porcine reproductive and respiratory syndrome viruses [5]. In particular, macrophages serve as a reservoir throughout HIV infection [3]. Importantly, macrophages also play a key role in avian viral infections including infectious bursal disease virus (IBDV) [6], avian influenza virus (AIV) [7], Newcastle disease virus (NDV) [8] and infectious bronchitis virus (IBV) [9]. However, the role of macrophages in ALV-J infection remains unclear.

In our previous study, we found that primary chicken monocyte-derived macrophages (MDM) were susceptible to ALV-J and infection resulted in expression of immune-related genes [10]. However, the number of genes we examined was too low to comprehensively map the involvement of immune host factors in an ALV-J infection. The goal of the current study was to examine host gene expression profile to improve our understanding of the relationship between macrophages and ALV-J during infection.

In this study, RNA-seq analysis platform and gene overexpression verification were employed to analyze chicken MDM gene expression after ALV-J infection. Our findings provide a comprehensive view of ALV-J immune escape and the host defense response to ALV-J infection in chicken macrophages.

## Materials and methods

### Animals and viruses

A total of 12 six-week-old specific-pathogen-free (SPF) White Leghorn chickens, half females and half males, were purchased from Guangdong DaHuaNong Animal Health Products Co., Ltd (Guangzhou, China) and housed under pathogen free conditions. Laboratory ALV-J strain SCAU-HN06 was kindly provided by Prof. Weisheng Cao, South China Agricultural University. All animal experiments were performed with approval and guidance from South China Agricultural University Institutional Animal Care and Use Committee.

### Culture of primary chicken MDM

Chicken primary MDM were cultured and identified according to previous studies [10, 11]. Briefly, peripheral blood mononuclear cells (PBMC) were isolated from blood obtained from SPF chickens using chicken lymphocyte separation medium (Solarbio, Beijing, China) according to the manufacturer’s instructions. The supernatant was removed and adherent cells were washed twice with PBS to remove thrombocytes, non-adherent lymphocytes and other semi-adherent cells after 6 h of incubation. These adherent cells were primarily chicken monocytes. Subsequently, fresh RPMI-1640 medium with 15% chicken serum, 100 U/mL penicillin and 100 mg/mL streptomycin were added to the remaining monocytes. Chicken monocytes were then cultured for 6 days to generate mature macrophage differentiation. The culture medium was changed every 2 days in order to ensure stable and consistent conditions.

### Detection of ALV-J replication in MDM

Chicken MDM were infected with a 10^5^ TCID_50_/mL of ALV-J strain SCAU-HN06. DNA, RNA and total proteins were extracted from the ALV-J infected MDM at 3, 6, 12, 24 and 36 h post-infection (hpi). RT-PCR was employed to detect the ALV-J replication using specific PCR primers H5/H7 [12]. Western blotting was performed with ALV-J envelope protein specific mouse antibody JE9 (kindly provided by Dr Aijian Qin, Yangzhou University, Yangzhou, China) and rabbit anti-β-actin antibody (Bioworld, Louis Park, USA) according to the method described previously [13]. IRDye 700DX-conjugated anti-rabbit IgG and IRDye 800-conjugated anti-mouse IgG (Rockland Immunochemicals, Limerick, PA, USA) was used as the secondary antibody. Membranes were visualized and analyzed with an Odyssey infrared imaging system (LI-COR Biosciences, Lincoln, NE, USA). ALV-J provirus was detected by PCR with primers H5/H7 using DNA template.

### Total RNA isolation

Total RNA for RNA sequencing (RNA-Seq) was isolated from pooled MDM (isolated and cultured from 12 SPF chickens) infected with ALV-J (10^5^ TCID_50_/mL) at 3 and 36 hpi using TRIzol reagent (Invitrogen, CA, USA). Samples were collected from two independent experiments. Non-infected MDM were used as a control group. Purity and quantity of total RNA were assessed using the NanoPhotometer^®^ spectrophotometer (Implen, CA, USA) and the Bioanalyzer 2100 system (Agilent Technologies, CA, USA). RNA degradation and contamination were further monitored using agarose gel electrophoresis.

### Library preparation for mRNA sequencing

After quality inspection, approximately 3 μg RNA per sample was used as input material for the RNA sample preparations. Briefly, ribosomal RNA was first removed using the Ribo-zero™ rRNA Removal Kit (Epicentre, WI, USA), and rRNA free residue was cleaned up by ethanol precipitation. Subsequently, sequencing libraries were generated using the rRNA-depleted RNA by NEBNext^®^ Ultra™ Directional RNA Library Prep Kit for Illumina^®^ (NEB, MA, USA) according to the manufacturer’s recommendations. First strand cDNA was synthesized with random hexamers and M-MuLV Reverse Transcriptase. Second strand cDNA synthesis was subsequently performed using DNA Polymerase I and RNase H. In the reaction buffer, dNTP containing dTTP were replaced with dUTP. Remaining overhangs were converted into blunt ends via the exonuclease and polymerase activities. After adenylation of 3′ ends of DNA fragments, NEBNext Adaptor with hairpin loop structure were ligated to prepare for hybridization activities. In order to select cDNA fragments of preferentially 150–200 bp in length, the library fragments were purified with AMPure XP system (Beckman Coulter, Beverly, USA). Then 3 μL USER Enzyme (NEB, Ipswich, MA, USA) was used with size-selected, adaptor-ligated cDNA at 37 °C for 15 min followed by 5 min at 95 °C before PCR. Then, PCR was performed with Phusion High-Fidelity DNA polymerase, universal PCR primers, and Index (X) Primers. At last, products were purified (AMPure XP system) and library quality was assessed on the Agilent Bioanalyzer 2100 system. The mRNA libraries were sequenced at the Novogene (Beijing, China) on an Illumina Hiseq 2000 platform.

### Data analysis of mRNA

Raw data (raw reads) of fastq format were first processed through in-house perl scripts. In this step, clean data (clean reads) were obtained by removing adapter sequences as well as reads containing poly-N and low quality reads. Therefore, only high quality data were analyzed and quality scores (Q20 and Q30) and GC content were subsequently calculated. All the following analyses were based on the clean data with high quality. Reads were mapped to the chicken genome assembly [14] using Tophat (v2.0.9). The mapped reads of each sample were assembled by both Scripture (beta2) and Cufflinks (v2.1.1) in a reference-based approach.

### Quantification of gene expression level

The FPKM (fragments per kilo-base of exon per million fragments mapped) was calculated based on the length of the fragments and reads count mapped to this fragment. Cuffdiff (v2.1.1) was used to calculate FPKM of coding genes in each sample. Gene FPKM were computed by summing the FPKM of transcripts in each gene group.

### Differential expression analysis

Cuffdiff software was used to provide statistical routines for determining differential expression in digital transcripts or gene expression data using a model based on the negative binomial distribution. In the present study, for differentially expressed genes (DEG), the threshold was q value < 0.05, log2 |(fold change)| ≥ 1 with an FPKM value no less than 10 in infected or uninfected samples.

### Gene ontology, and pathway analysis

DEG were subjected to Gene Ontology (GO) categorization and Kyoto Encyclopedia of Genes and Genomes (KEGG) pathway analysis using the Database for Annotation, Visualization, and Integrated Discovery (DAVID) version 6.8 [15].

### Validation of gene expression in RNA-seq by quantitative real-time PCR (qPCR)

Total RNA was extracted from ALV-J-infected (10^5^ TCID_50_/mL) and uninfected MDM at 3 hpi and 36 hpi using RNAiso reagent (TaKaRa, Japan). For gene expression analysis, cDNA synthesis of mRNA was performed using a PrimeScript RT Reagent Kit (Perfect Real Time) (TaKaRa, Japan) according to the manufacturer’s protocol. The qPCR primers were designed using the NCBI Primer BLAST program [16] and were based on published target sequences (Additional file 1A) [17–20]. The GAPDH gene was used as an internal control. qPCR was performed on a Bio-Rad CFX96 Real-Time Detection System using iTaqTM Universal SYBR^®^ Green Supermix Kit reagents (Bio-Rad, CA, USA) according to the manufacturer’s specifications. Data analyses were performed using the 2^−ΔΔCt^ method.

### Transfection of up-regulated DEG and detection of their function on ALV-J replication

MDM were cultured in 12-well plates and transfected with 1 μg plasmids including K60, IRG1, OASL, CH25H, CISH, EX-FABP, IL4I and SOCS3 using Lipofectamine 3000 reagent, respectively. EGFP was used as a control. After incubation for 4 h, Lipofectamine 3000 transfection reagent was removed, and the cells were replenished with RPMI-1640 medium with 15% chicken serum. 24 h later, the transfected MDM were infected with 10^5^ TCID_50_/mL of ALV-J strain SCAU-HN06. At 3 hpi, ALV-J replication level was analyzed by Western blot and qPCR. The primers used in the construction of these plasmids are summarized in Additional file 1B.

### Statistical analyses

Statistical comparisons were performed using GraphPad Prism 5 (GraphPad Software Inc., San Diego, CA, USA). The results are presented as the mean ± SEM. Two-way ANOVA analysis was used to analyze the statistical significance among multiple groups and unpaired Student’s *t*-test between two groups. Statistical significance is indicated by *p* values of > 0.05 (non-significant, ns), < 0.05 (*), 0.01 (**) or 0.001(***).

### Raw data information

The sequencing data obtained from RNA-Seq were released to the GEO database under the accession numbers GSE103207.

## Results

### Detection of ALV-J in chicken MDM

**Figure 1:**
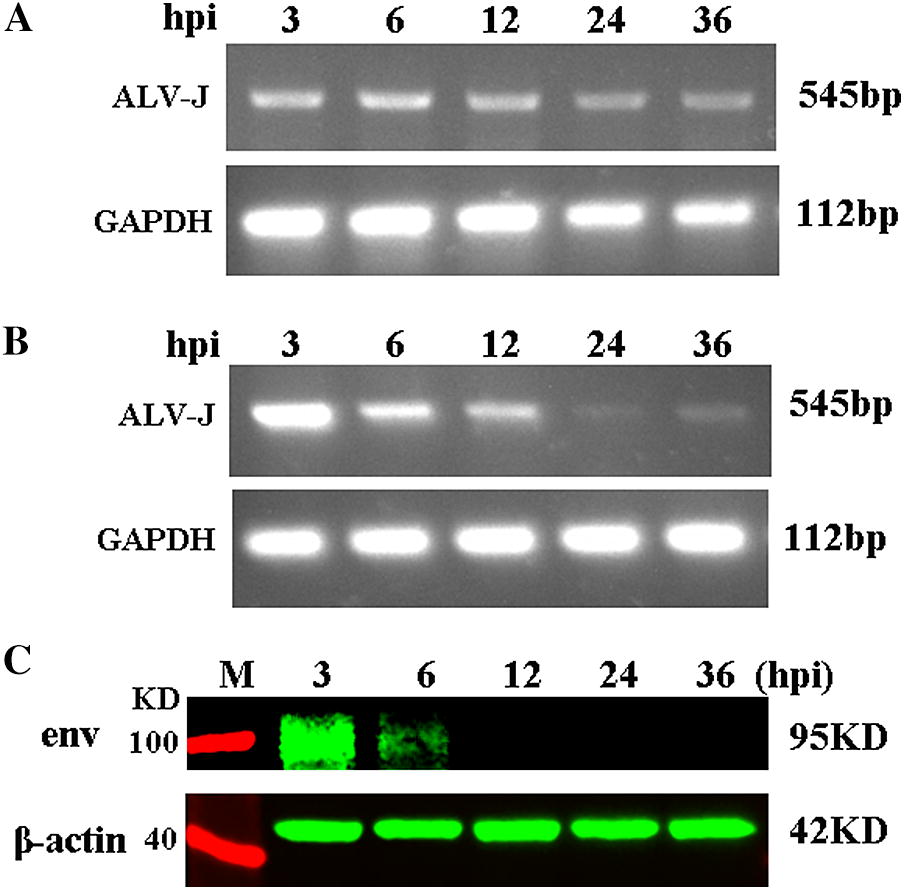
**Detection of ALV-J replication in chicken MDM from 3 hpi to 36 hpi. A** PCR detection with DNA template and ALV-J specific primer. All samples extracted from 3 hpi to 36 hpi produced specific 545 bp fragment. **B** RT-PCR detection with cDNA template and ALV-J specific primer. **C** Western blotting analysis shows that samples extracted from 3 hpi produced obvious specific ALV-J envelope protein blots, but the protein blots became weaker at 6 hpi and tended to disappear at 12 to 36 hpi.

ALV-J infections of MDM resulted in either genome integration or reverse transcription into cDNA during 3-36 hpi (Figure 1A). The rate of ALV-J replication was very high at 3 hpi and gradually decreased from 6 to 36 hpi (Figure 1B). Furthermore, the viral envelope protein was detectable by Western blotting at 3 and 6 hpi but not at later times. Similarly, *env* gene expression at 3 hpi was greater than that at 6 hpi (Figure 1C). These results demonstrate that the replication rate of ALV-J was extremely high at 3 hpi but low after 6 hpi, ALV-J replication might be inhibited in chicken MDM.

### Differentially expressed genes after ALV-J infection in chicken MDM

The Illumina HiSeq 2000 platform produced 814 412 986 raw reads. After discarding adaptor and low-quality sequences, we obtained 794 083 068 clean reads (119.19 Gb). The clean reads were mapped onto the chicken reference genome (Gallus_gallus-4.0), and the mapping rate of each library ranged from 79.29 to 83.27% (Additional file 2).

**Figure 2:**
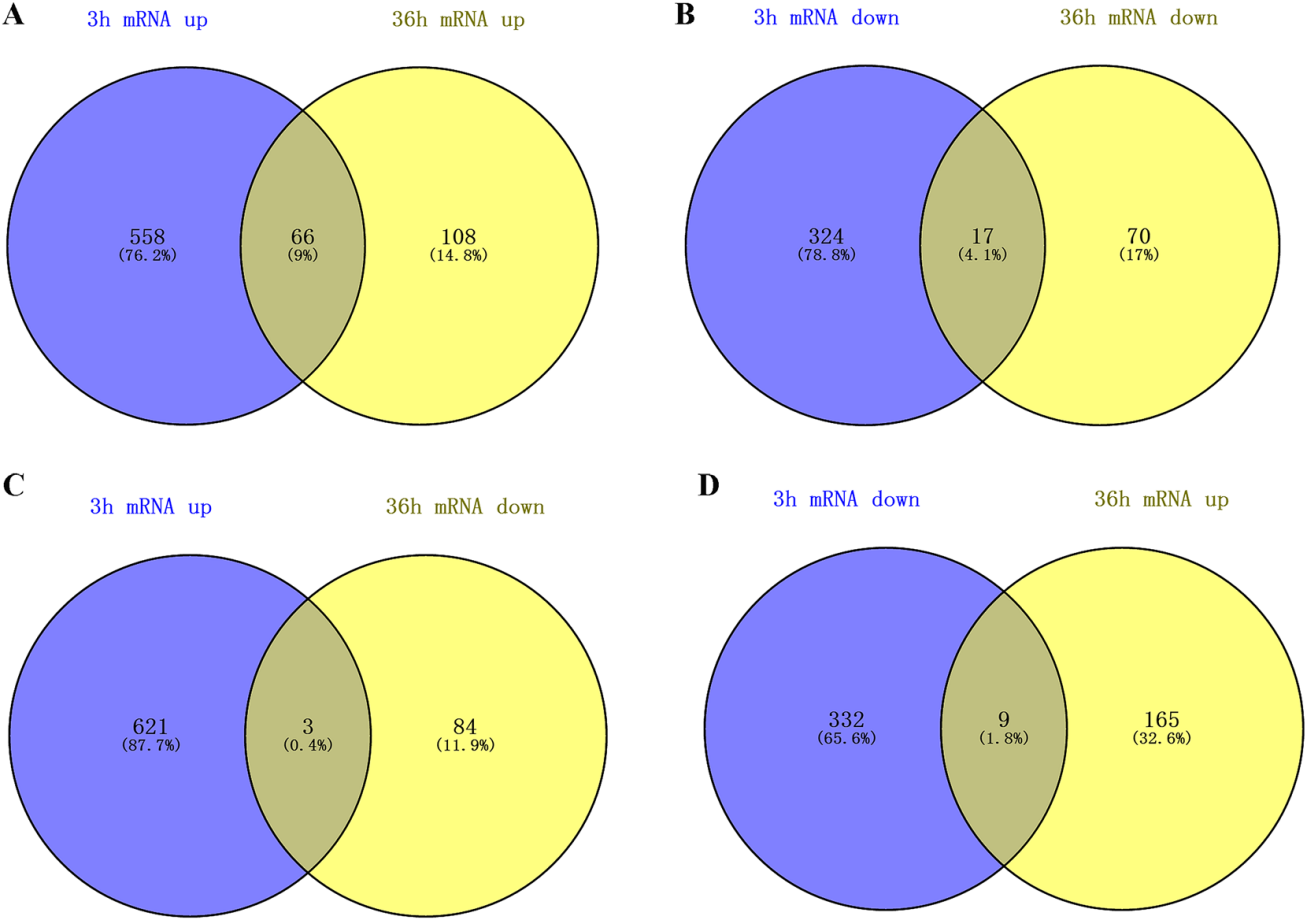
**DEG in chicken MDM infected with ALV-J at 3 hpi and 36 hpi.** Venn diagrams of up-regulated DEG (**A**) and down-regulated DEG (**B**) at 3 hpi and 36 hpi from chicken MDM. **C** Venn diagrams of up-regulated DEG at 3 hpi and down-regulated DEG at 36 hpi in chicken MDM. **D** Venn diagrams of down-regulated DEG at 3 hpi compared up-regulated DEG at 36 hpi in chicken MDM.

We found that at 3 and 36 hpi, 558 and 108 DEG were uniquely up-regulated, and 324 and 70 were uniquely down-regulated in ALV-J infected MDM, respectively. There were 66 up-regulated and 17 down-regulated DEG in common at the two time points (Figures 2A and B). In addition, three DEG, *OASL*, *FKBP51* and *MCF2*, were up-regulated at 3 hpi and down-regulated at 36 hpi (Figure 2C). Nine DEG were down-regulated at 3 hpi but up-regulated at 36 hpi (Figure 2D). More details of the DEG are shown in Additional file 3.

### GO annotation of DEG after ALV-J infection in chicken MDM

**Figure 3:**
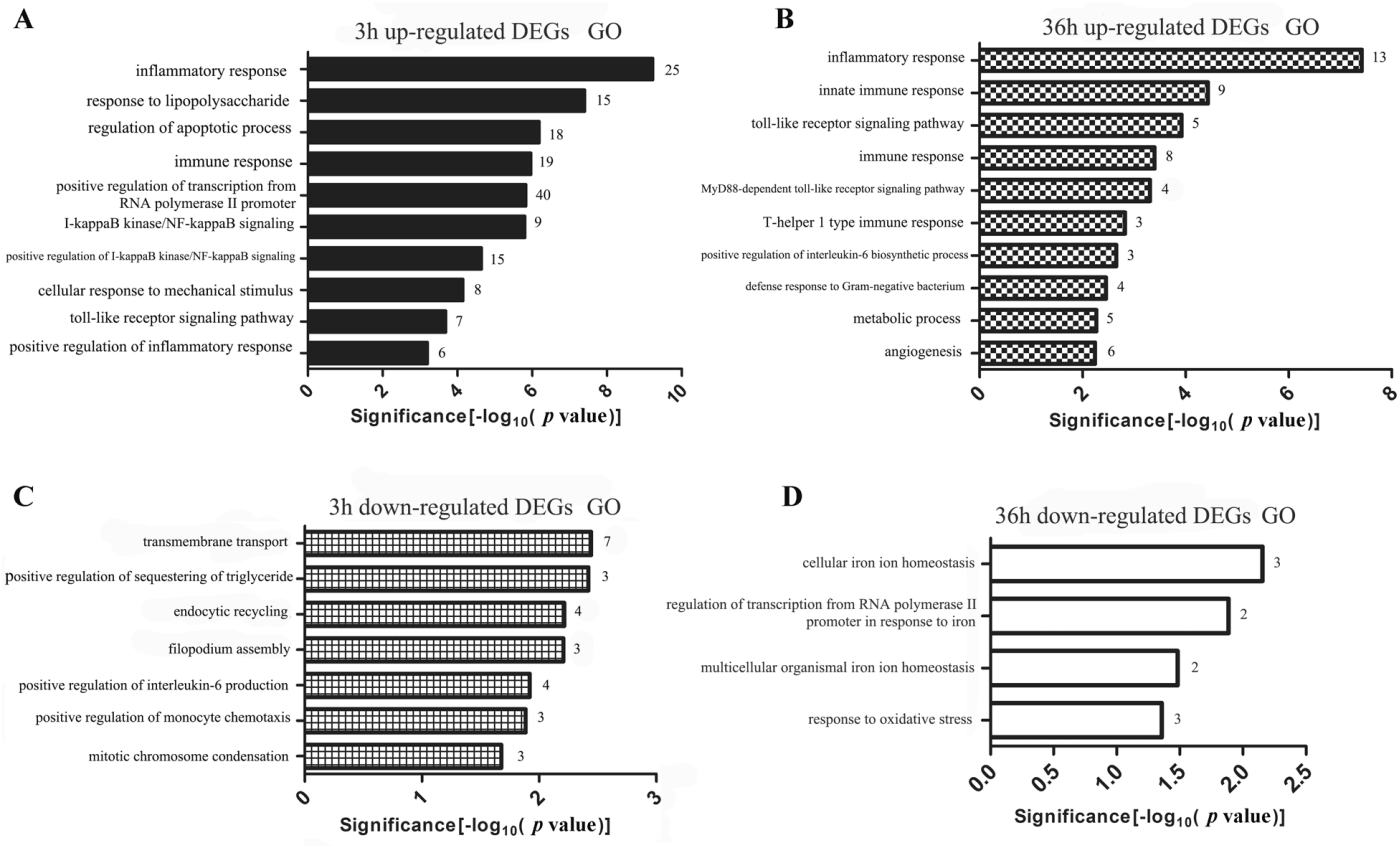
**Gene ontology (GO) terms analysis of DEG expressed in ALV-J-infected MDM.** Representative GO terms of up-regulated DEG in the ALV-J-infected MDM at **A** 3 hpi and **B** 36 hpi. Down-regulated DEG enriched in the representative GO terms of ALV-J-infected MDM at **C** 3 hpi and **D** 36 hpi.

The GO biological process analysis shows that the up-regulated DEG were mainly enriched for immune-related terms but down-regulated DEG were not (Figure 3). For the up-regulated DEG at 3 hpi, the top three significant GO terms were inflammatory response, response to lipopolysaccharide and regulation of apoptotic process (Figure 3A). The top three significant GO terms for up-regulated DEG at 36 hpi were inflammatory response, innate immune response and Toll-like receptor signaling pathway (Figure 3B). Down-regulated DEG at 3 hpi were significantly enriched for transmembrane transport, endocytic recycling and positive regulation of interleukin-6 production (Figure 3C). The five down-regulated DEG at 36 hpi included *HMOX1*, *SLC11A1*, *SLC40A1*, *GAB1* and *SLC25A4* were significantly enriched on four GO terms (Figure 3D). More details of the DEG involved in GO enrichment analysis can be found in Additional file 4.

### Pathway analysis of DEG after ALV-J infection in chicken MDM

**Figure 4:**
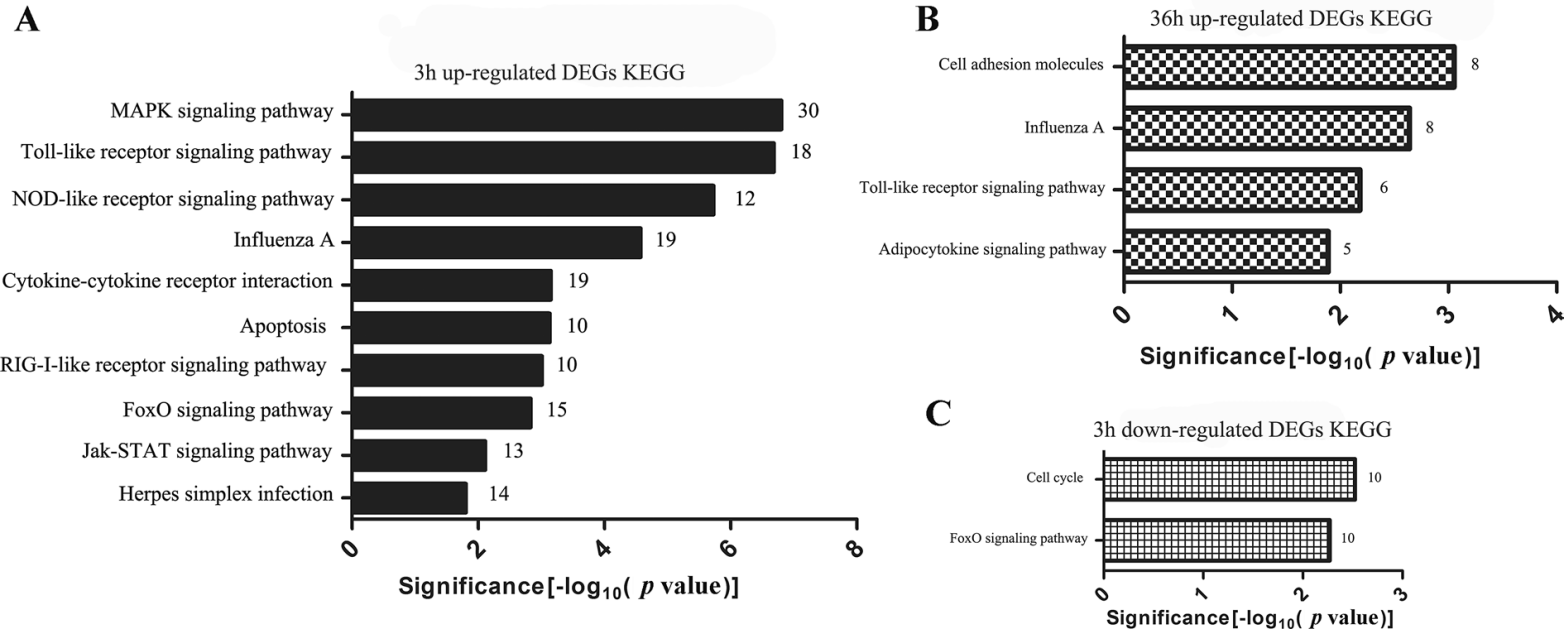
**KEGG pathways in ALV-J-infected MDM.** KEGG pathways of up-regulated DEG expressed in ALV-J-infected MDM at **A** 3 hpi and **B** 36 hpi. **C** Down-regulated DEG enriched in KEGG pathways of ALV-J-infected MDM at 3 hpi.

KEGG analysis illustrated that up-regulated DEG induced by ALV-J in MDM at 3 hpi were involved in immune-related pathways including MAPK signaling, Toll-like, NOD-like, RIG-I-like and Jak-STAT signaling pathway, and etc. (Figure 4A). Up-regulated DEG identified at 36 hpi were significantly enriched in cell adhesion molecules, influenza A, Toll-like receptor and adipocytokine signaling pathway (Figure 4B). However, just two pathways were significantly enriched by the down-regulated DEG at 3 hpi (Figure 4C). Moreover, down-regulated DEG at 36 hpi did not enrich any pathway. More details of the DEG involved in KEGG enrichment analysis can be found in Additional file 5.

### More immune-related DEG were induced by ALV-J at 3 hpi than at 36 hpi

**Figure 5:**
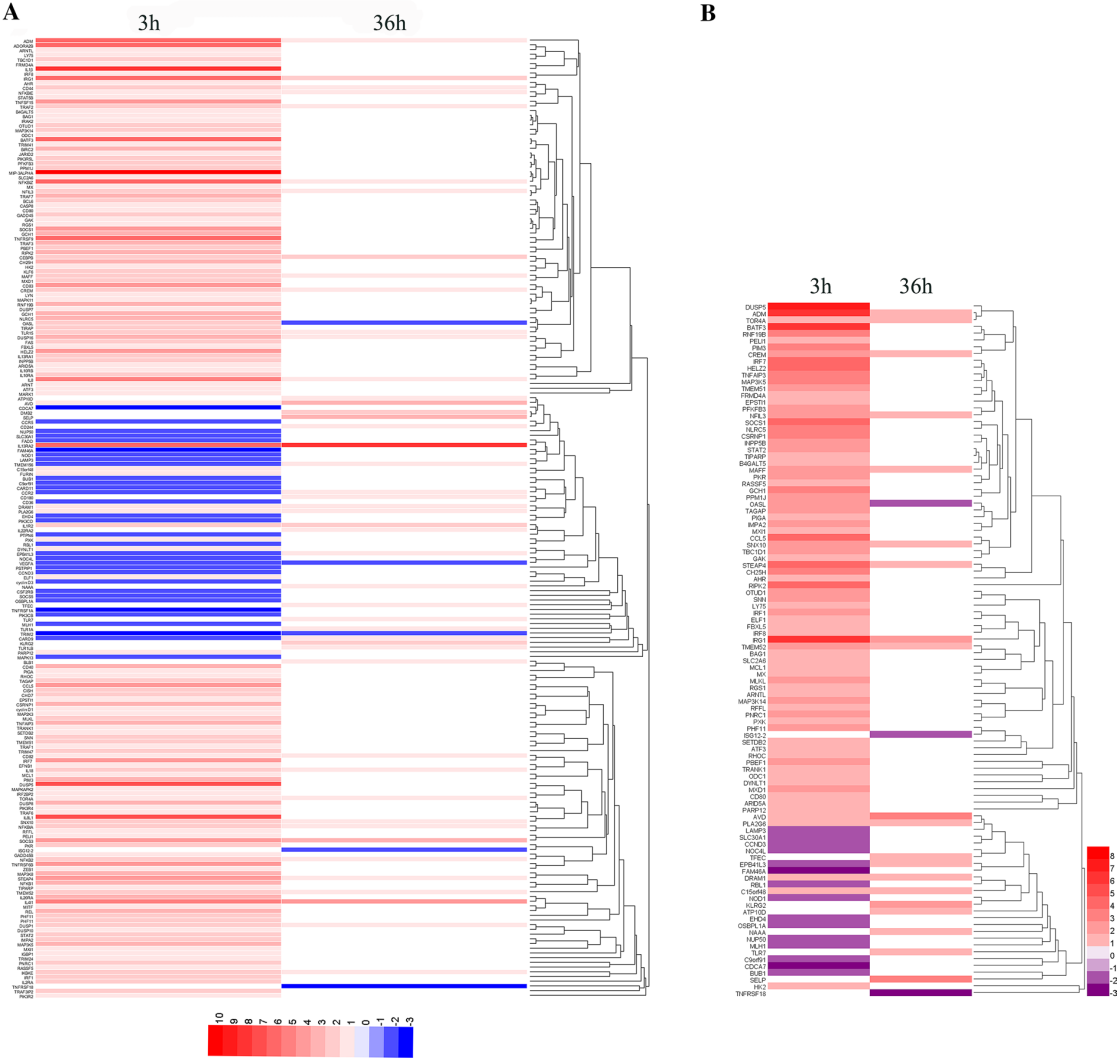
**Heatmap of immune-related DEG at different time points after ALV-J infection.** DEG with similar expressed patterns were clustered and are displayed in a heatmap format. Color intensity corresponds to relative expression level normalized according to log_2_ fold change. **A** Selected immune-related DEG from 3 hpi and 36 hpi. Red, up-regulated DEG; blue, down-regulated DEG. **B** ISG expression in MDM at 3 hpi and 36 hpi. Red, up-regulated ISG; purple, down-regulated ISG.

The immune-related genes were selected according to gene function annotation. A greater number of immune-related DEG were found at 3 hpi than at 36 hpi, and most of these immune-related DEG were up-regulated by ALV-J infection at 3 hpi (Figure 5A). According to published studies [21–23], 94 and 23 differentially expressed interferon-stimulated genes (ISG) were identified in ALV-J-infected MDM at 3 hpi and 36 hpi, respectively (Additional file 6). Similarly, the expression of most ISG (79) was significantly increased at 3 hpi, especially *IRG1*, *RIPK2*, *CH25H*, *IRF7* and etc. (Figure 5B).

### RNA-seq data matched the qPCR data

**Figure 6:**
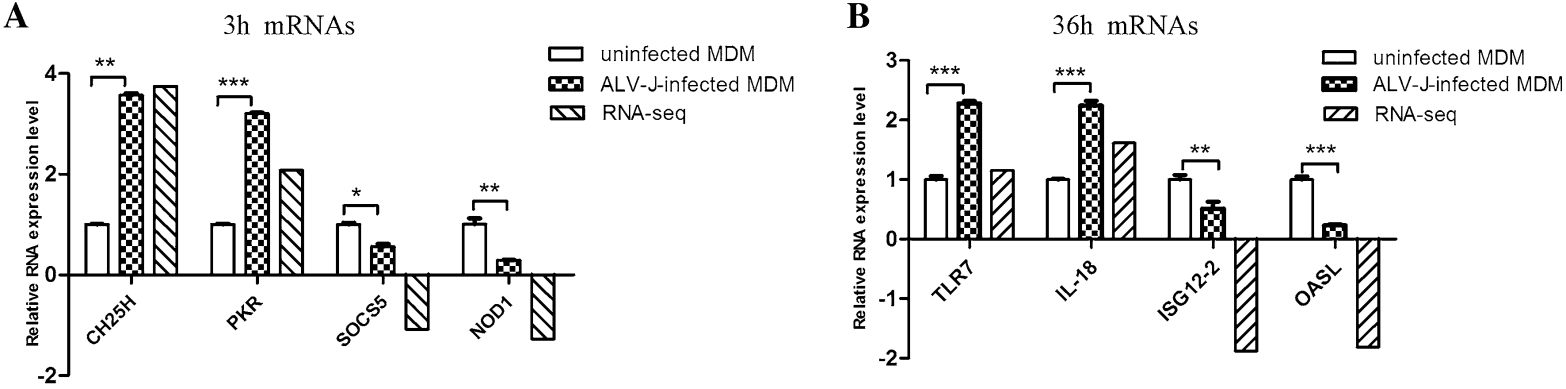
**Validation of RNA-Seq data by qPCR. DEG were selected at (A) 3 hpi and (B) 36 hpi.** qPCR results were represented using relative expression value. RNA-seq value is log_2_(foldchange) values of DEG. *p < 0.05, **p < 0.01, ***p < 0.001.

To validate the RNA-Seq results, we chose eight immune-related DEG for qPCR analysis. These included *CH25H*, *PKR*, *SOCS5*, *NOD1*, *TLR7*, *IL*-18, *ISG12*-2 and *OASL.* The qPCR data matched the RNA-Seq results and both methods indicate similar trends for these eight genes (Figure 6).

### Overexpression of *K60*, *IRG1*, *OASL* and *CH25H* inhibits ALV-J replication

**Figure 7:**
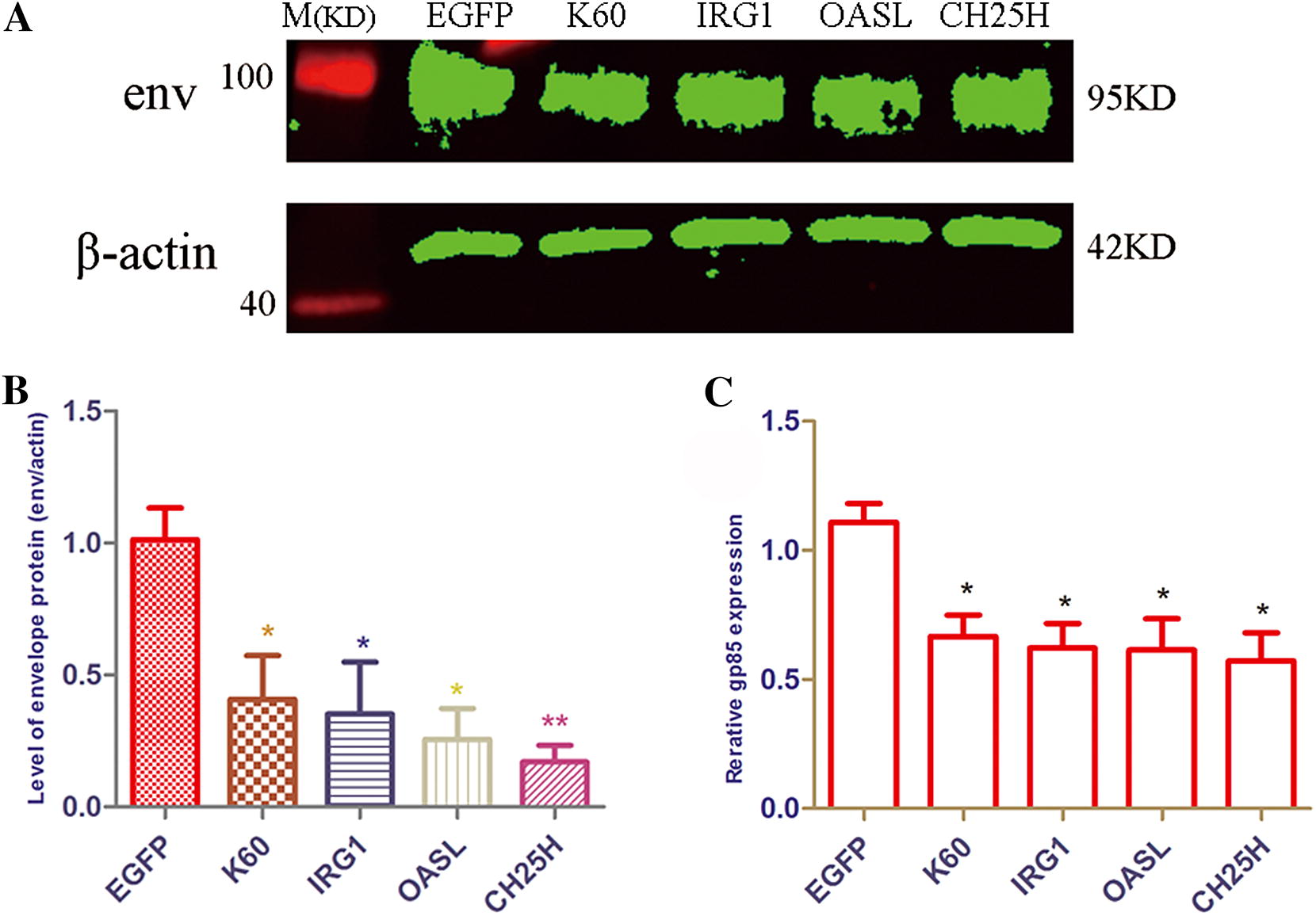
**Overexpression of** ***K60***, ***IRG1***, ***OASL*** **and** ***CH25H*** **could inhibit ALV-J replication.** MDM cells were transfected with pCMV-K60, pCMV-IRG1, pCMV-OASL, pCMV-CH25H and infected with ALV-J strain SCAU-HN06 at 24 post-transfection. MDM cells transfected with pCMV-EGFP as a control. **A** SCAU-HN06 strain envelop protein was measured by Western blot at 3 hpi. **B** The level of SCAU-HN06 strain envelop protein was analyzed by Image Studio Ver 5.2 (Odyssey Fc). **C** The expression of SCAU-HN06 *env* gene was measured by qPCR at 3 hpi. *p < 0.05, **p < 0.01.

Overexpression of *K60*, *OASL*, *CH25H* and *IRG1* significantly decreased ALV-J replication at the protein (Figures 7A and B) and mRNA (Figure 7C) levels in chicken MDM cells at 3 hpi when compared to the control group (EGFP).

### Overexpression of *CISH*, *EX*-*FABP*, *IL4I1* and *SOCS3* promotes ALV-J replication

**Figure 8:**
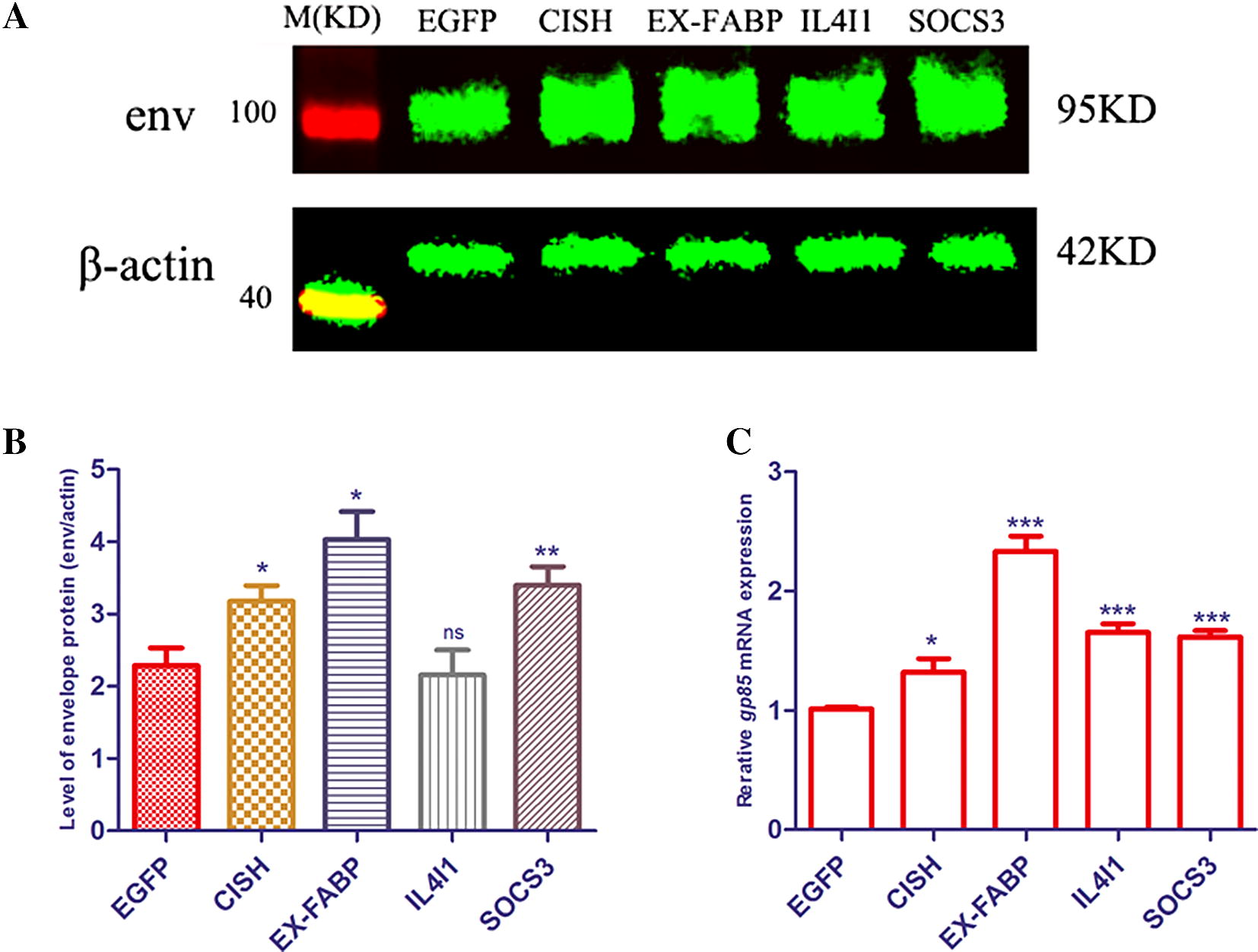
**Overexpression of** ***CISH***, ***EX*-*FABP***, ***IL4I1*** **and** ***SOCS3*** **could enhance ALV-J replication.** MDM cells were transfected with plasmids including *EGFP*, *CISH*, *EX*-*FABP*, *IL4I1* and *SOCS3* and infected with SCAU-HN06 at 24 post-transfection. **A**, **B** The level of SCAU-HN06 strain envelop protein was detected by Western blot at 3 hpi and analyzed by Image Studio Ver 5.2 (Odyssey Fc). (C) The expression of SCAU-HN06 *env* gene was detected by qPCR at 3 hpi. *p < 0.05, **p < 0.01.

Overexpression of *CISH*, *EX*-*FABP* and *SOCS3* significantly increased the expression of ALV-J *env* gene at protein levels (Figures 8A and B) and mRNA levels (Figure 8C) in chicken MDM cells at 3 hpi. However, overexpression of *IL4I1* only significantly increased the expression of the ALV-J *env* gene at the mRNA level, but there was no difference at the protein level (Figure 8).

## Discussion

### Host anti-ALV-J candidates

As an avian retrovirus, ALV-J has been studied for many years although many interesting scientific problems such as tumorigenesis, immunosuppression and immune responses induced by ALV-J infection are still not understood [1]. In our previous studies, we found that chicken MDM are susceptible to ALV-J infection [10]. In the present study, we observed that ALV-J replication in MDM was active at 3 hpi, but inhibited from 6 to 36 hpi. It is reported that recombinant chicken IFN-α as well as the ISG, CCCH type zinc finger antiviral protein (ZAP), could inhibit ALV-J replication in DF1 cells [24, 25]. So, we speculated that ISG may also resist ALV-J replication in chicken MDM.

ALV-J infection induced most immune-related DEG in MDM at 3 hpi (Tables 1 and 2). Strikingly, the expression of 79 ISG including *CH25H*, *PKR, OASL*, *Mx*, and etc. were significantly increased in ALV-J-infected MDM at 3 hpi. ISG exert numerous antiviral effector functions by targeting almost any step in the virus life cycle [26]. For example, CH25H broadly inhibited growth of enveloped viruses including VSV, HSV, HIV and the acutely pathogenic viruses EBOV, RVFV and RSSEV by converting cholesterol to 25-hydroxycholesterol (25HC) [27]. Additionally, 18 up-regulated ISG were identified in ALV-J MDM at 36 hpi. All of these up-regulated ISG in ALV-J-infected MDM at 3 and 36 hpi might serve as candidates resisting ALV-J infection.

**Table 1:** **The DEG listed involved in the pattern recognition receptor pathway and Jak-STAT signaling pathway in ALV-J-infected MDM**

Gene ID	Gene name	Biological processes	log_2_ (fold change) 3 hpi, 36 hpi	Source of DEG
*Pattern recognition receptor pathway* ① Toll-like receptor signaling pathway; ② NOD-like receptor signaling pathway; ③ RIG-I-like receptor signaling pathway; ④ Cytosolic DNA-sensing pathway				
ENSGALG00000007015	CD40	①	3.89259, –	3 hpi
ENSGALG00000015474	CD80	①	1.74169, –	3 hpi
ENSGALG00000011389	TRAF3	①, ③	3.76105, –	3 hpi
ENSGALG00000007932	TRAF6	①, ②, ③	1.27085, –	3 hpi
ENSGALG00000009014	TRAF2	③	2.74956, 1.13451	3 hpi and 36 hpi
ENSGALG00000000951	CCL5	①, ④	4.20314, –	3 hpi
ENSGALG00000013356	IKBKE	①, ③, ④	2.38933, 1.06398	3 hpi and 36 hpi
ENSGALG00000014297	IRF7	①, ③, ④	4.51047, –	3 hpi
ENSGALG00000000534	IL1β	①, ②, ④	8.57939, –	3 hpi
ENSGALG00000011668	K60 (IL8L1)	①, ②, ③	7.00993, –	3 hpi
ENSGALG00000026098	IL8 (IL8L2)	①, ②, ③	5.96016, 1.67045	3 hpi and 36 hpi
ENSGALG00000008612	MAPK11(p38Beta)	①, ②, ③	1.56955, –	3 hpi
ENSGALG00000004735	MAP2K3(MKK3)	①	1.62121, –	3 hpi
ENSGALG00000007356	MAP3K8	①	3.52264, –	3 hpi
ENSGALG00000012304	NFKB1	①, ②, ③, ④	3.76626, –	3 hpi
ENSGALG00000003428	PIK3R2	①	1.30282, –	3 hpi
ENSGALG00000021573	PIK3R5L	①	2.7941, 1.12565	3 hpi and 36 hpi
ENSGALG00000026167	PIK3R5	①	2.77632, 1.13829	3 hpi and 36 hpi
ENSGALG00000001077	TIRAP	①	2.04202, –	3 hpi
ENSGALG00000013861	TNFAIP3 (A20)	②	3.42548, –	3 hpi
ENSGALG00000017186	BIRC2	②	3.67041, –	3 hpi
ENSGALG00000007874	IL18	②, ④	2.72387, 1.6161	3 hpi and 36 hpi
ENSGALG00000015899	RIPK2	②	4.27785, –	3 hpi
ENSGALG00000021325	RIPK3	②	3.04709, –	3 hpi
ENSGALG00000017485	TLR1A	①	–, 1.47044	36 hpi
ENSGALG00000027093	TLR1B	①	–, 1.10129	36 hpi
ENSGALG00000016590	TLR7	①	–, 1.15464	36 hpi
ENSGALG00000008166	TLR15		2.10934, 1.89122	3 hpi and 36 hpi
ENSGALG00000027864	NFKBIA		2.10466, 1.14704	3 hpi and 36 hpi
ENSGALG00000005653	NFKB2		2.8071, 1.41077	3 hpi and 36 hpi
Jak-STAT signaling pathway				
ENSGALG00000002260	CISH (CIS)		2.58373, –	3 hpi
ENSGALG00000003282	STAT5B		1.29572, –	3 hpi
ENSGALG00000007158	SOCS1		4.22098, –	3 hpi
ENSGALG00000027786	SOCS3		4.08564, 3.14348	3 hpi and 36 hpi
ENSGALG00000010016	SOCS5		−1.07644, –	3 hpi

**Table 2:** **DEG with significant changes in expression at 3 hpi and 36 hpi**

Gene ID	Gene name	3 hpi FPKM (J/NC), log_2_(foldchange)	36 hpi FPKM (J/NC), log_2_(foldchange)
ENSGALG00000003003	MIP-3α (CCL20)	(2519.22/2.23788), 10.1366	–
ENSGALG00000000534	IL-1β	(3039.04/7.94481), 8.57939	–
ENSGALG00000005069	PTGS2 (COX-2)	(179.952/0.74049), 7.92492	–
ENSGALG00000005693	iNOS (NOS2)	(1289.62/6.00488), 7.7466	–
ENSGALG00000011668	K60(IL8L1)	(2559.75/19.8609), 7.00993	–
ENSGALG00000014182	ADORA2B	(144.072/1.51003), 6.57606	–
ENSGALG00000016286	CXorf21	(717.697/7.58374), 6.56432	–
ENSGALG00000006352	CH25H	(623.719/46.4632), 3.74674	–
ENSGALG00000016919	IRG1	(5752.22/67.2951), 6.41747	(77.9823/13.1535), 2.5677
ENSGALG00000015346	NFKBIZ	(1250.44/16.1651), 6.27342	(12.6029/6.1146), 1.04343
ENSGALG00000006337	K123	(491.778/8.8756), 5.79202	(12.9952/1.95007), 2.73638
ENSGALG00000000081	IL4I1	(119.384/3.37829), 5.14318	(23.0818/0.794182), 4.86114
ENSGALG00000017184	MMP7	–	(76.752/4.11625), 4.2208
ENSGALG00000009963	LYZ (lysozyme)	(821.232/74.5905), 3.46073	(5524.5/62.4031), 6.46808
ENSGALG00000024011	EX-FABP	(679.788/93.0983), 2.86826	(15805/51.4835), 8.26205
ENSGALG00000027716	HPS5	(459.873/127.921), 1.84599	(3600.15/52.3945), 6.1025
ENSGALG00000013723	OASL	(56.9902/11.452), 2.31511	(6.0525/21.2826), − 1.81407
ENSGALG00000000947	FKBP51	(59.357/13.3894), 2.14832	(11.6466/48.2111), − 2.04946
ENSGALG00000006562	MCF2 (DBL)	(20.0391/9.42061), 1.08892	(6.14223/13.7507), − 1.16268

In addition to the above ISG, some DEG have significant changes in expression at 3 hpi and 36 hpi (Table 2). MIP-3α, macrophage inflammatory protein-3 alpha, is responsible for the chemo-attraction of dendritic cells, and effector and memory B and T cells [28]. Moreover, MIP-3α exhibited anti-microbial and anti-HIV activities [28–30]. IL-1β is produced primarily by activated macrophages and possess multiple and diverse properties in their response to infection [31, 32]. Thus, host damage following infection induces macrophage secretion of a variety of inflammatory mediators including IL-1 and NO that activate anti-pathogenic microorganism defense mechanisms [2]. NO production is primarily catalyzed by iNOS and is a part of innate host defenses [33]. ALV-J infection in MDM at 3 hpi increased expression of the two orthologues of chicken IL-8, K60 (IL8L1) and IL8 (IL8L2) [34, 35]. IL-8 is a potent chemo-attractant and activator of macrophages [36]. Furthermore, IL-8 has been shown to attract and activate T lymphocytes [37, 38], which would aid in raising an immunologically specific response against ALV-J. Immune response gene 1 (*IRG1*) was originally identified as a highly inducible gene in murine macrophages following LPS stimulation [39]. The role of IRG1 in the course of virus infection has not been extensively reported. IRG1 was identified as an ISG with antiviral effects against different neurotropic viruses [40]. OASL has been found to broadly inhibit the replication of viruses such as swine fever virus, RSV and HCV through a variety of mechanisms [41–43]. Lysozyme is a differentiation marker for macrophage, and is activated during macrophage differentiation [44]. We found that lysozyme expression increased incrementally from 3 hpi to 36 hpi in ALV-J-infected MDM. This result reminded us that ALV-J infection could stimulate chicken macrophage maturation. Lysozyme is a cornerstone of innate immunity due to its direct antimicrobial activity through peptidoglycan hydrolysis and immune regulatory functions [45]. Interestingly, lysozyme also possesses antiviral properties [46, 47]. *NFKBIZ* encodes the protein IκBz and is known as a partner of NFκB that regulates innate host defense factors [48].

Accordingly, the host genes *MIP*-*3α*, *IL*-*1β*, *iNOS*, *K60*, *IRG1*, *CH25H*, *OASL*, *lysozyme* and *NFKBIZ* served as restriction factor candidates against ALV-J infection in chicken macrophages. We further selected several of the anti-ALV-J candidates for verification. Exactly, the experiments in vitro show that overexpression of *K60*, *IRG1*, *CH25H*, and *OASL* could significantly decrease ALV-J replication in MDM at 3 hpi (Figure 7).

### The survival strategy of the ALV-J in MDM

Initially, we found that ALV-J infection activated many pattern recognition receptors (PRR) pathways including Toll-like receptors (TLR), RIG-I-like receptors (RLR), NOD-like receptors (NLR) and cytosolic DNA-sensing pathway at 3 hpi (Figure 4). Up to now, the specific innate sensors responding to ALV were unknown [1]. We speculated that ALV-J should theoretically be recognized by PRR such as TLR, RLR, IFI16, and cGAS, similar to HIV [1]. However, no functional PRR such as TLR3, TLR4, TLR7 or MDA5 were induced in ALV-J-infected MDM at 3 hpi. Only TLR15 was up-regulated by ALV-J at 3 hpi (Table 1). Therefore, we speculate that chicken macrophages lack functional PRR for ALV-J to escape host immune attack at the early stage of infection. Indeed, it has been reported that other retroviruses use this strategy to achieve immune escape in macrophages [49]. Interestingly, the expression of TLR1, TLR7 and TLR15 was significantly increased in ALV-J-infected MDM at 36 hpi (Table 1). As a result, ALV-J replication was inhibited and weak innate immune responses were induced at 36 hpi. At this infection stage, ALV-J may be recognized by TLR1, TLR7 and TLR15.

The Jak-STAT pathway is a major signaling pathway in the function of immune cells and is activated by cytokines and growth factors [50]. In this study, many negative feedback regulators of cytokine signaling mediated by this pathway were identified. *CISH*, *SOCS1* and *SOCS3* were significantly induced in ALV-J-infected MDM at 3 hpi and enriched on the Jak-STAT signaling pathway (Table 1). CISH and suppressor of cytokine signaling (SOCS) family proteins are Jak-STAT inhibitors, including 8 members, CISH, SOCS1, SOCS2, SOCS3, SOCS4, SOCS5, SOCS6 and SOCS7 [51]. CISH, SOCS1, SOCS2 and SOCS3 are the best characterized SOCS family members [52]. CISH is induced by cytokines that activate STAT5 and block the STAT binding to cytokine receptors. SOCS1 binds to the Jaks and inhibits catalytic activity, while SOCS3 binds to Jak-proximal sites on cytokine receptors and inhibits Jak activity [52]. It has been reported that SOCS3 enhances HIV-1 replication in macrophages by inhibiting antiviral IFN-β signaling [53]. The *SOCS3* expression was also significantly increased in ALV-J-infected MDM (Table 1). Further verification found that overexpression of *CISH* and *SOCS3* promoted ALV-J replication in MDM at 3 hpi (Figure 8).

We speculated that ALV-J infection inhibited the Jak-STAT pathway via inducing expression of *CISH*, *SOCS1* and *SOCS3*. Indeed, the key factors such as Jak and STAT in Jak-STAT pathway were not remarkably induced by ALV-J infection in MDM. The role of SOCS5 has not been well identified during viral infection. However, a novel role for SOCS5 has been found to restrain the early phase of influenza A infection by inhibiting EGFR activity [54]. Coincidently, *SOCS5* expression was inhibited by ALV-J in MDM at 3 hpi (Table 1).

In addition, the expression of *NFKBIA* and *TNFAIP3* (A20) were significantly increased in ALV-J-infected MDM at 3 hpi (Table 1). The IκBα protein is encoded by *NFKBIA* and is an important negative regulatory factor in the NF- κB pathway [55]. The protein TNFAIP3 is known as a powerful suppressor of cytokine signaling and innate antiviral pathways, and it can inhibit the activity of NF-κB and NF-κB-mediated inflammatory responses [56, 57]. *TNFAIP3* deficiency in myeloid cells and lung epithelial cells could protect against influenza A virus infection [58, 59]. We speculated that *NFKBIA* and *TNFAIP3* were significantly induced by ALV-J infection in MDM to down-regulate cytokines in macrophages, resulting in viral persistence in the host. Altogether, *NFKBIA*, *TNFAIP3*, *CISH*, *SOCS1* and *SOCS3* were considered as a counterbalance to the antiviral immune responses induced by ALV-J infection in chicken MDM at 3 hpi.

*IL4I1*, *PTGS2 (COX*-*2)* and *EX*-*FABP* significantly changed expression at 3 hpi and 36 hpi (Table 2). We also found that overexpression of *IL4I1* and *EX*-*FABP* could enhance ALV-J replication in chicken MDM at 3 hpi (Figure 8). IL4I1 is an immunosuppressive enzyme and primarily expressed in professional antigen-presenting cells and it inhibits T-cell proliferation and activation [60, 61]. COX-2 is one of the important mediators of inflammation in response to viral infection, which contributes to viral replication and this has been shown for HCV [62], HBV [63], dengue virus [64] and cytomegalovirus [65]. The *EX*-*FABP* gene encodes an extracellular fatty acid binding protein and it is significantly induced by *Salmonella enteritidis* infections in chickens [66]. This protein may provide fatty acids for mitochondrial respiration during infection. *EX*-*FABP* expression is enhanced after treatments with inflammatory stimuli and is repressed by anti-inflammatory agents, behaving as an acute phase and constitutively expressed survival protein [67]. *EX*-*FABP* was robustly induced in ALV-J-infected MDM at 36 hpi and therefore may provide protection for MDM after the chemokine and cytokine production induced by ALV-J at 3 hpi. There is a rule that the virus is lost when the cells die. Consequently, induction of EX-FABP at 36 hpi may be a strategy of ALV-J to live in harmony with chicken macrophages at the late stages of infection. Consequently, *EX*-*FABP*, *IL4I1* and *COX*-*2* together with *NFKBIA*, *TNFAIP3*, *CISH*, *SOCS1* and *SOCS3* enable ALV-J survival in chicken macrophages.

### A balanced view for the interactions between ALV-J and host immune response

The low levels of ALV-J replication in MDM at 36 hpi was accompanied with fewer immune-related DEG involved in host defense responses. Superficially, we could conclude that ALV-J replication was inhibited at 6-36 hpi due to robust host immune responses induced at 3 hpi. However, in HIV-related studies, the conventional host immune response does not contain HIV-1 replication and even contributes by increasing virus replication through immune activation [68]. Moreover, viruses can proactively hide in the host to evade the host immune elimination and HIV typically establishes latency within the macrophage [69]. Based on our findings, it is also possible that ALV-J is capable of escaping from host immune responses and establishing latency in chicken MDM after 3 h of viral infection. According to the above analyses, we should take a balanced view to consider the interactions between ALV-J and host immune response. Additional studies are needed to elucidate the mechanisms of ALV-J immune evasion and host defense responses in chicken macrophages.

In summary, gene expression profiling analysis in chicken MDM infected with ALV-J provides insights into the mechanisms underlying the host immune responses and ALV-J immune escape. Strong immune responses were induced by ALV-J infection in MDM at 3 hpi. We found that numerous differentially expressed genes such as *MIP*-*3α*, *IL*-*1β*, *iNOS*, *K60*, *IRG1*, *CH25H*, *NFKBIZ*, *lysozyme* and *OASL* were involved in host defense of ALV-J infection. ALV-J countered host immune attacks by inhibiting the expression of functional PRR and facilitating expression of Jak-STAT pathway inhibitors. These results provide valuable insights into the antagonism between host antiviral immune responses and ALV-J infection.

## Additional files

**Additional file 1.** **List of primers used in the study.**

**Additional file 2.** **RNA-Seq data statistics.**

**Additional file 3.** **DEG details at 3 hpi and 36 hpi after ALV-J infection in MDM.**

**Additional file 4.** **GO terms and the information of DEG involved in these GO terms at 3 hpi and 36 hpi after ALV-J infection in MDM.**

**Additional file 5.** **KEGG pathways and the information of DEG involved in these KEGG pathways at 3 hpi and 36 hpi after ALV-J infection in MDM.**

**Additional file 6.** **ISG induced by ALV-J infection in chicken MDM at 3 hpi and 36 hpi.**
