# Supplementary material for: A balanced game: chicken macrophage response to ALV-J infection
Source: Vet Res. 2019 Mar 6;50:20. doi: 10.1186/s13567-019-0638-y (PMC6404279; doi:10.1186/s13567-019-0638-y)
Supplement: Supplementary file 1 — Additional file 1. List of primers used in the study. [file 13567_2019_638_MOESM1_ESM.docx]

**Additional file 1A qPCR primers used in verification of avian genes.**

| Target | Primer | Sequence(5’-3’) |
| --- | --- | --- |
| TLR7 | Forward | TCTGGACTTCTCTAACAACA |
|  | Reverse | AATCTCATTCTCATTCATCATCA |
| PKR | Forward | CCTCTGCTGGCCTTACTGTCA |
|  | Reverse | AAGAGAGGCAGAAGGAATAATTTGCC |
| SOCS5 | Forward | TTAGCCCCCGGTATGACTGA |
|  | Reverse | TGCGCGACTGTAGACAAAGT |
| NOD1 | Forward | GCGATGCAGGAATTGGAAAA |
|  | Reverse | TGTGAAAAGAACCGTA TGAGGGA |
| CH25H | Forward | AATCCAGCCGCAGAGCTATC |
|  | Reverse | CAGCTCTGGAGCTATCACCG |
| IL-18 | Forward | GTTCGATTTAGGGAAGGAGAAGT |
|  | Reverse | GTCTTCTTCCTCAAAGGCCAAG |
| ISG12-2 | Forward | GGAATTGCAAGAGGTTCTC |
|  | Reverse | CCCATTTCTTGTAGAGTAGC |
| OASL | Forward | CACGGCCTCTTCTACGACA |
|  | Reverse | TGGGCCATACGGTGTAGACT |
| GAPDH | Forward | GAACATCATCCCAGCGTCCA |
|  | Reverse | CGGCAGGTCAGGTCAACAAC |

**Additional file 1B The primers used in the construction of DEGs plasmids**

| Target | Primer | Sequence(5’-3’) |
| --- | --- | --- |
| K60 | Forward | GGGGggtctctagtg ATGATGGGCAAGGCTGTAGCTG |
|  | Reverse | GCCGggtctcgtggg TTAGGATGCAGTCTTATTATTGGTGT |
| IRG1 | Forward | GGGGggtctctagtg ATGATTCTGGATACCCTTGGAG |
|  | Reverse | GCCGggtctcgtggg TTATGAGTTGGAATGTTCAACGTG |
| OASL | Forward | GGGGggtctctagtg ATGGGGTTGGAGAGCGTGAG |
|  | Reverse | GCCGggtctcgtggg AGGAGGGCACGCAGCGTCTGGG |
| CH25H | Forward | GGGGggtctctagtg ATGAACTGCAGCGTGCGGG |
|  | Reverse | GCCGggtctcgtggg TTAACTAGGATGTGACTCTGTG |
| CISH | Forward | GGGGggtctctagtg ATGATCCTCTGCGTCCC |
|  | Reverse | GCCGggtctcgtggg TCAGAGCTGGAAGGGGTATT |
| EX-FABP | Forward | GGGGcgtctcgagtg ATGAGGACGCTGGCACTGA |
|  | Reverse | GCCGggtctcgtggg CTACACTTCATCAACGCTGC |
| IL4I1 | Forward | GGGGggtctctagtg ATGGCTGCGATGGTTCTCTTCCAA |
|  | Reverse | GCCGggtctcgtggg TCAGAGCTCTCCCTTCTCCACGA |
| SOCS3 | Forward | GGGGggtctctagtg ATGGTCACCCACAGCAAG |
|  | Reverse | GCCGggtctcgtggg TTAGAGGGGGGCATCGTAC |

Transformed commercial pCMV vector was purchased from SIDANSAI, China.
