# Supplementary material for: A balanced game: chicken macrophage response to ALV-J infection
Source: Vet Res. 2019 Mar 6;50:20. doi: 10.1186/s13567-019-0638-y (PMC6404279; doi:10.1186/s13567-019-0638-y)
Supplement: Supplementary file 2 — Additional file 2. RNA-Seq data statistics. [file 13567_2019_638_MOESM2_ESM.docx]

RNA-Seq data statistics

| Sample name | Raw reads | Clean reads | Clean bases | Total mapped |
| --- | --- | --- | --- | --- |
| NC3h-1 | 102742884 | 100089308 | 15.01G | 82.8% |
| NC3h-2 | 115905336 | 113028716 | 16.95G | 80.35% |
| J3h-1 | 96950168 | 94769226 | 14.22G | 81.28% |
| J3h-2 | 101042058 | 97945402 | 14.69G | 79.29% |
| NC36h-1 | 105863240 | 103164224 | 15.47G | 83.27% |
| NC36h-2 | 91083018 | 88792738 | 13.32G | 83.16% |
| J36h-1 | 108291818 | 105599624 | 15.84G | 83.23% |
| J36h-2 | 92534464 | 90693830 | 13.6G | 82.88% |
